# 2,3-Dimethoxycinnamic Acid from a Marine Actinomycete, a Promising Quorum Sensing Inhibitor in *Chromobacterium violaceum*

**DOI:** 10.3390/md22040177

**Published:** 2024-04-16

**Authors:** Yanqun Li, Wenping Ding, Jiajia Yin, Xingyu Li, Xinpeng Tian, Zhihui Xiao, Fazuo Wang, Hao Yin

**Affiliations:** 1 CAS Key Laboratory of Tropical Marine Bio-Resources and Ecology, South China Sea Institute of Oceanology, Chinese Academy of Sciences, Guangzhou 510301, China; liyanqun20@mails.ucas.ac.cn (Y.L.); dingwenping19@mails.ucas.ac.cn (W.D.); yinjiajia22@mails.ucas.ac.cn (J.Y.); bsy_lixingyu@126.com (X.L.); xinpengtian@scsio.ac.cn (X.T.); xzh@scsio.ac.cn (Z.X.); wangfazuo@scsio.ac.cn (F.W.); 2 University of Chinese Academy of Sciences, Beijing 100049, China; 3 Southern Marine Science and Engineering Guangdong Laboratory (Guangzhou), Guangzhou 511458, China; 4 Sanya Institute of Ocean Eco-Environmental Engineering, Sanya 572025, China

**Keywords:** *Nocardiopsis metallicus* SCSIO 53858, quorum sensing inhibitor, 2,3-dimethoxycinnamic acid, *Chromobacterium violaceum*, molecular docking

## Abstract

An ethyl acetate extract of a marine actinomycete strain, *Nocardiopsis mentallicus* SCSIO 53858, isolated from a deep-sea sediment sample in the South China Sea, exhibited anti-quorum-sensing (QS) activity against *Chromobacterium violaceum* CV026. Guided by the anti-QS activity, a novel active compound was isolated and purified from the extract and was identified as 2,3-dimethoxycinnamic acid (2,3-DCA) through spectral data analysis. At a concentration of 150 μg/mL, 2,3-DCA exhibited robust inhibitory effects on three QS-regulated traits of *C*. *violaceum* CV026: violacein production, swarming motility, and biofilm formation, with inhibition rates of 73.9%, 65.9%, and 37.8%, respectively. The quantitative reverse transcription polymerase chain reaction results indicated that 2,3-DCA can disrupt the QS system in *C*. *violaceum* CV026 by effectively suppressing the expression of QS-related genes, including *cviR*, *vioA*, *vioB*, and *vioE*. Molecular docking analysis revealed that 2,3-DCA hinders the QS system by competitively binding to the same binding pocket on the CviR receptor as the natural signal molecule N-hexanoyl-L-homoserine lactone. Collectively, these findings suggest that 2,3-DCA exhibits promising potential as an inhibitor of QS systems, providing a potential solution to the emerging problem of bacterial resistance.

## 1. Introduction

Quorum sensing (QS) is a collective behavior that influences the expression of specific genes in microorganisms, regulating their physiological activities and facilitating self-coordination among microbial populations through the detection of signal molecules released by these populations when cell density reaches a particular threshold. The QS regulatory mechanism is closely tied to the virulence and pathogenicity of pathogenic microorganisms [1,2]. Quorum sensing inhibitors (QSIs) offer promising alternatives to traditional antibiotics by effectively regulating the QS behavior of bacteria, thereby reducing their pathogenicity and virulence. Unlike traditional antibiotics that block and interfere with the biosynthetic pathways of pathogenic bacteria, QSIs operate through a distinct mechanism, diminishing the likelihood of selective pressure and resulting in a low probability of drug resistance [3]. The discovery of QSIs not only enhances our understanding of microbial community adaptation to the environment, but also serves as a valuable reference for the development of anti-drug-resistance medications.

*Chromobacterium violaceum* has emerged as a model organism for investigating bacterial communication in Gram-negative bacteria, boasting a distinctive QS mechanism contingent upon the type of signaling molecule released [4]. The production of easily detectable and quantifiable violacein pigments by QS-regulated vio operons offers a straightforward method for screening potential QS regulators and/or inhibitors. This QS-regulated pigment is not only easy to visualize and quantify but also holds the potential for the development of biosensors [5,6].

Over 70% of the Earth’s surface is covered by oceans, which harbor microbial resources with unique metabolic mechanisms resulting from their distinct ecological environments—characterized by high pressure, high salt, low temperature, hypoxia, and oligotrophy. A growing number of chemical drugs with therapeutic efficacy have been derived from the secondary metabolites of marine microorganisms, emphasizing the importance of marine microorganisms as a resource for natural drugs [7,8,9]. Researchers are progressively directing their focus towards bacterial QS systems to screen for QSIs from metabolites of marine microorganisms, with the aim of developing novel antibacterial drugs [10,11,12,13]. Marine actinomycetes, known for their valuable contributions, serve as abundant sources of novel secondary metabolites characterized by complex structures and unique activities, making them exceptional candidates for the screening of potential QSIs [14,15,16]. 

Recently, using the model of *C*. *violaceum* CV026, we conducted a screening of anti-QS active strains derived from marine actinomycetes isolated from deep-sea sediment samples in the South China Sea and made a noteworthy discovery that the crude extract of strain SCSIO 53858 exhibited a significant reduction in violacein production. The ongoing study aimed to investigate the anti-QS activity of the potential QSI-producing strain SCSIO 53858; the active compounds were isolated and purified through activity-guided fractionation, the chemical structures of the active compounds were identified through spectral data analysis, their anti-QS activity was characterized, and the possible mechanism of the active compounds was elucidated. This study will provide valuable insights into the environmental adaptation mechanism of strain SCSIO 53858 and contribute to the development of novel antimicrobial agents to combat drug resistance.

## 2. Results and Discussion

### 2.1. Isolation and Structural Elucidation of QSIs from N. mentallicus SCSIO 53858

**Figure 1:**
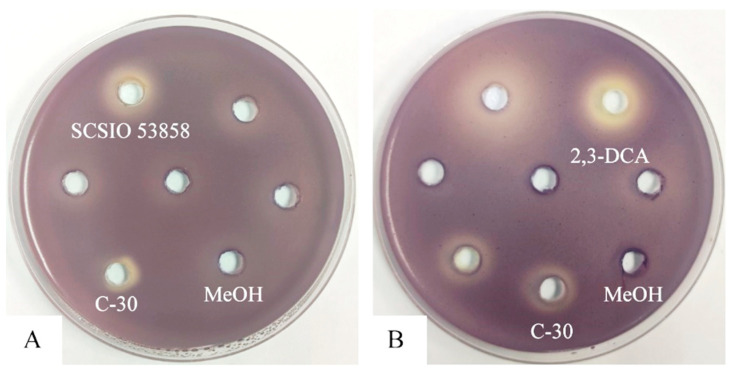
Anti-QS disc assay in *C*. *violaceum* CV026: (**A**) 200 mg/mL of the crude extract of strain SCSIO 53858; (**B**) 10 mg/mL of 2,3-DCA. Furanone C-30 was used as positive control. Methanol (MeOH) was used as a negative control.

**Figure 2:**
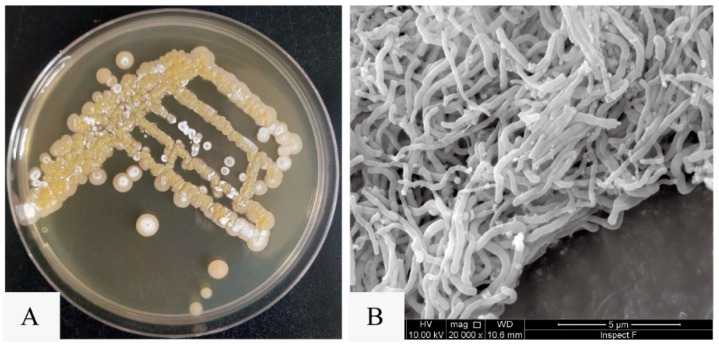
The colony morphology and mycelial morphology of *N*. *mentallicus* SCSIO 53858. (**A**) Colonies of *N*. *mentallicus* SCSIO 53858 grown on MA medium for 5 days. (**B**) SEM image of *N*. *mentallicus* SCSIO 53858 after growth on MB medium for 3 days at 28 °C.

Utilizing the violacein assay (Figure 1A), we successfully isolated a marine actinomycete strain with anti-QS activity from marine sediments collected in the South China Sea. Designated as strain SCSIO 53858, this microorganism was cultured in marine 2216 medium at 28 °C for 5 days and presented as an aerobic actinobacterium, displaying yellow-brown substrate mycelium and white aerial mycelium with straight spore chains, each approximately 0.5 μm in diameter (Figure 2). Additionally, through 16S rRNA gene sequence analysis with a phylogenetic tree constructed by the neighbor-joining method (Figure S1), the strain SCSIO 53858 was identified and named as *N*. *mentallicus* SCSIO 53858 [17].

**Figure 3:**
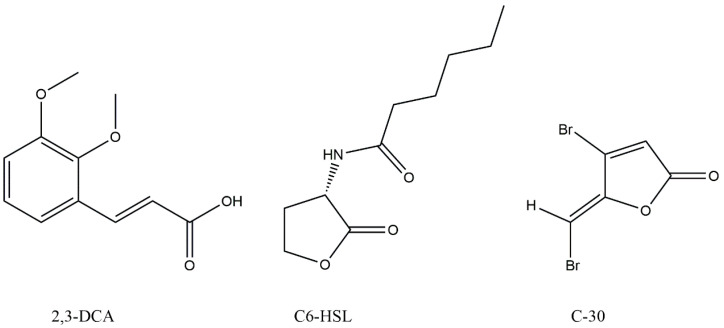
Chemical structures of 2,3-dimethoxycinnamic acid (2,3-DCA), N-hexanoyl-L-homoserine lactone (C6-HSL), and furanone C-30 (C-30).

In pursuit of generating extracts suitable for the exploration of active constituents, *N. mentallicus* SCSIO 53858 underwent solid-state fermentation, conducted concurrently across numerous 1 L vessels. After extraction, a total of 17.6 g of ethyl acetate extract was obtained. Based on the violacein assay, the compound (27.7 mg) with anti-QS activity was further purified on Octadecylsilyl (ODS) column chromatography, silica gel column chromatography, and preparative high-performance liquid chromatography (HPLC). The data obtained from nuclear magnetic resonance (NMR) spectroscopy (Figures S2 and S3) were as follows: ^1^H NMR (700 MHz, methanol-*d*_4_, *δ*, ppm, TMS): *δ*_H_ 7.60 (d, *J* = 15.9 Hz, 1H), 7.17 (d, *J* = 1.9 Hz, 1H), 7.06 (dd, *J* = 8.2, 1.9 Hz, 1H), 6.80 (d, *J* = 8.2 Hz, 1H), 6.35 (d, *J* = 15.9 Hz, 1H), 3.89 (s, 3H), 3.76 (s, 3H); ^13^C NMR (176 MHz, methanol-*d*_4_, *δ*, ppm, TMS): *δ*_C_ 169.9, 150.8, 149.5, 147.0, 127.8, 124.2, 116.6, 115.3, 111.8, 56.6, 52.1. By comparison with literature NMR values [18], the structure of this compound was identified as 2,3-dimethoxycinnamic acid (2,3-DCA) (Figure 3).

2,3-DCA used in the study on QS inhibitory activity was isolated from the crude extract of *N. mentallicus* SCSIO 53858. As shown in Figure 1B, 2,3-DCA exhibited an inhibitory effect on violacein production in *C*. *violaceum* CV026, while the minimum inhibitory concentration (MIC) of the 2,3-DCA against *C*. *violaceum* CV026 was determined to be over 500 μg/mL. Although 2,3-DCA is a common cinnamic acid derivative, to the best of our knowledge, only its ability to promote the formation of long and entangled wormlike micelles has been reported to date [19,20]. The isolation of 2,3-DCA from *N*. *metallicus* and its QS inhibitory effects on *C*. *violaceum* have not been reported. Notably, our study marks the first observation of the inhibitory effect of 2,3-DCA at subinhibitory concentrations on the bacterial QS system.

### 2.2. Inhibition of QS-Regulated Virulence Factor and Bacterial Motility of 2,3-DCA in C. violaceum CV026

**Figure 4:**
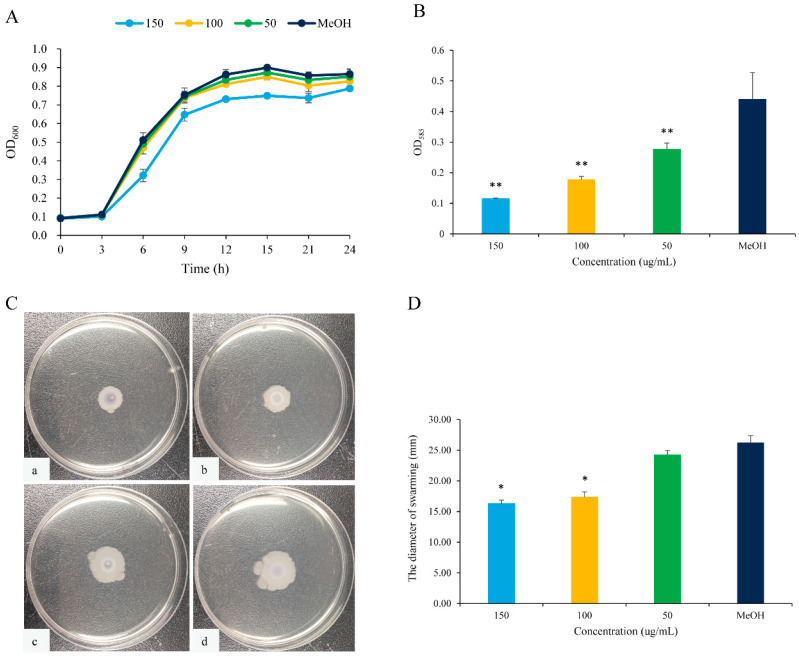
QS inhibition of 2,3-DCA in *C*. *violaceum* CV026. (**A**) The growth curves of *C*. *violaceum* CV026 treated with 150, 100, and 50 μg/mL 2,3-DCA for 24 h in Luria-Bertani broth (LB). (**B**) The effects of 2,3-DCA on violacein production of *C*. *violaceum* CV026 treated with 150, 100, and 50 μg/mL 2,3-DCA for 24 h in LB broth. (**C**) Bacterial movements on swarming media of *C*. *violaceum* CV026 treated with 150 μg/mL 2,3-DCA (**a**), 100 μg/mL 2,3-DCA (**b**), 50 μg/mL 2,3-DCA (**c**), and MeOH (**d**). (**D**) Measurement of the swarming motility. MeOH was used as a negative control. Data are shown as the mean ± standard deviation (SD) (*n* = 3) with error bars representing standard errors. * *p* < 0.05 and ** *p* < 0.01 compared with the control group.

The anti-QS effects of 2,3-DCA against *C*. *violaceum* CV026 were evaluated by measuring the secretion of QS-regulated virulence factors. As depicted in Figure 4, 2,3-DCA effectively reduced the violacein production and inhibited swarming motility without exhibiting inhibitory effects on the growth of *C*. *violaceum* CV026 within the concentration range of 50 to 150 μg/mL. 

The expression of the purple pigment violacein, encoded by the *vio* operon, is regulated by the QS system in *C*. *violaceum* [21,22]. A concentration-dependent reduction in violacein production was observed in *C*. *violaceum* CV026 treated with 2,3-DCA below 150 μg/mL, while the growth curve of *C*. *violaceum* CV026 displayed no significant changes compared to the control group (Figure 4A,B). The inhibition rates for violacein production were 37.3% at 50 μg/mL, 59.8% at 100 μg/mL, and 73.9% at 150 μg/mL (*p* < 0.05), supporting the observation that 2,3-DCA can inhibit the QS system of *C*. *violaceum* CV026.

Furthermore, swarming motility appears to be an intrinsically linked surface and cell density phenomenon and controlled by QS [23]. The swarming motility of *C*. *violaceum* CV026 was diminished by 2,3-DCA. As illustrated in Figure 4C,D, the surface colonization during swarming of *C*. *violaceum* CV026 decreased as the concentration of 2,3-DCA increased. Consequently, compared to the control group, the swarming inhibition rates were 33.7% at 100 μg/mL and 37.8% at 150 μg/mL (*p* < 0.05). 

The inhibition of violacein production and swimming motility by 2,3-DCA suggests some potential applications, warranting further evaluation.

### 2.3. Inhibition of Biofilm Formation of 2,3-DCA in C. violaceum CV026

**Figure 5:**
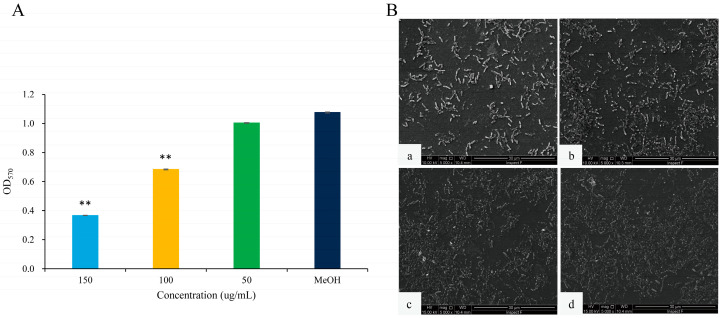
The effects of 2,3-DCA on biofilms of *C*. *violaceum* CV026. (**A**) Inhibition of biofilm production. Data are shown as the mean ± SD (*n* = 3) with error bars representing standard errors. ** *p* < 0.01 compared with the control group. (**B**) SEM images of *C*. *violaceum* CV026 biofilms treated with 150 μg/mL 2,3-DCA (**a**), 100 μg/mL 2,3-DCA (**b**), 50 μg/mL 2,3-DCA (**c**), and MeOH (**d**).

The formation of biofilms is one of the main causes of antibiotic resistance [24]. *C*. *violaceum* can produce highly structured and dense biofilms, primarily attributed to QS [25]. The inhibitory effects of 2,3-DCA at sub-MIC concentrations on *C*. *violaceum* CV026 biofilm formation were evaluated using the crystal violet assay. Compared to the control group, the biofilms were markedly reduced by 36.5% and 65.9% after exposure to 2,3-DCA at concentrations of 100 μg/mL and 150 μg/mL, respectively (Figure 5A).

The scanning electron microscopy (SEM) images of the biofilms provide further insights into the impact of 2,3-DCA on *C*. *violaceum* CV026 biofilm (Figure 5B). At test concentrations of 100 μg/mL (Figure 5B(b)) and 150 μg/mL (Figure 5B(a)), the bacterial biofilms were visibly disrupted, while the bacterial cells appeared to hold their integrity. In comparison, the control group treated with MeOH showed a dense and protective biofilm structure (Figure 5B(d)). The SEM images were consistent with the crystal violet assay results, supporting the conclusion that 2,3-DCA can effectively reduce the biofilm formation of *C*. *violaceum* CV026.

### 2.4. Anti-QS Mechanism Exploration of 2,3-DCA in C. violaceum CV026

**Figure 6:**
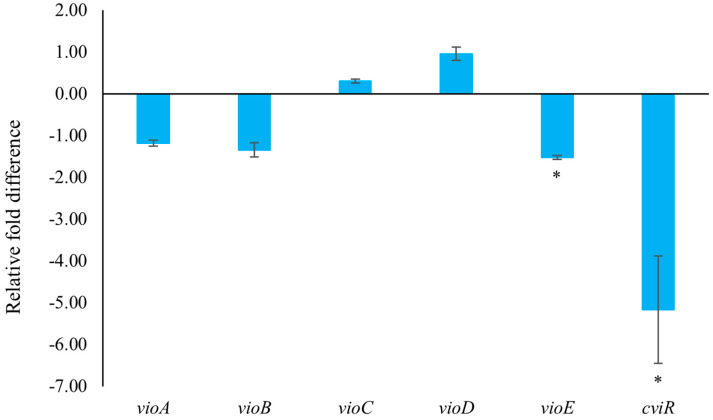
The effect of 2,3-DCA on the gene expression of *C*. *violaceum* CV026. Data are shown as the mean ± SD (*n* = 3) with error bars representing standard errors. * *p* < 0.05.

In *C*. *violaceum*, violacein is encoded by the *vio* operon (including *vioA*, *vioB*, *vioC*, *vioD*, and *vioE*), whose expression is regulated by QS-controlled systems comprising the CviI-CviR family of transcriptional regulators. The binding of the CviR-AHL complex to the promoter site of *vioA* triggers the expression of the *vio* operon, leading to the synthesis of the violacein [22]. *C*. *violaceum* CV026 is a mini-T5-mutant of the wild-type strain (*C*. *violaceum* 31532) This mutant lacks the *cviI* gene, which encodes the N-acyl homoserine lactone (AHL) synthase, thereby only allowing violacein production in response to externally supplied AHL signal molecules [5,26,27]. To assess the impact of 2,3-DCA on QS-related gene expression, quantitative reverse transcription polymerase chain reaction (qRT-PCR) technology was employed. The result clearly demonstrated that treatment with 150 μg/mL 2,3-DCA significantly inhibited the expression of QS-related genes (Figure 6). Specifically, 2,3-DCA treatment significantly inhibited the expression of key QS gene *cviR* and violacein-related gene *vioE* and inhibited violacein-related genes *vioA* and *vioB*. Conversely, 2,3-DCA treatment enhanced violacein-related genes *vioC* and *vioD*. The structure of violacein consists of a 5-hydroxyindole, an oxindole, and a 2-pyrrolidone. The biosynthesis of violacein involves two independent process pathways, including the enzymatic process catalyzed by five enzymes, VioA-E, and the alternative nonenzymatic oxidative decarboxylation reactions. The flavoenzyme VioA and the heme protein VioB work in conjunction to oxidize and dimerize L-tryptophan to the nascent product indole-3-pyruvic acid imine dimer. In the presence of VioE, the intermediate instead undergoes indole rearrangement to yield prodeoxyviolacein acid. Flavin-dependent oxygenase VioD hydroxylates one indole ring at the 5-position to yield proviolacein, and flavin-dependent oxygenase VioC then acts on the other indole ring at the 2-position to create the oxindole and complete violacein formation (Figure S4) [28,29]. Moreover, inhibition of VioA or VioB leads to total depletion of the violacein intermediate prodeoxyviolacein acid, and VioE is regarded as a key enzyme that “switches” the biosynthetic pathway through an intramolecular 1,2-shift of an indole ring; any alteration in the expression of these genes leads to a reduction in the production of purple pigment synthesis in *C*. *violaceum*, consequently reducing the bacteria’s toxicity [28,29,30,31]. The observed changes in gene expression suggest that the anti-QS ability of 2,3-DCA may arise from its interaction with QS-related proteins or DNA molecules, thereby disrupting the QS signaling pathways. 

**Figure 7:**
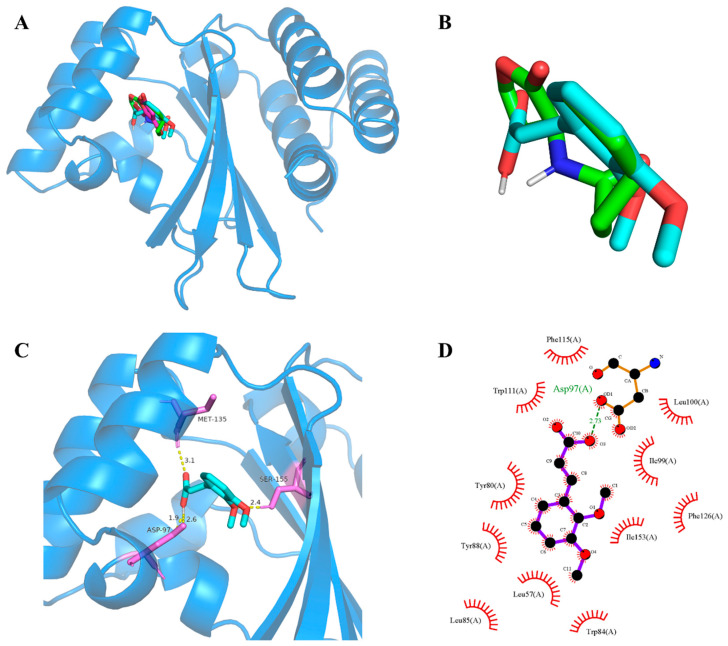
CviR-2,3-DCA docking and interaction profiling. (**A**) Docked complex of the CviR receptor protein with natural ligand C6-HSL, positive control C-30, and tested compound 2,3-DCA. CviR, C6-HSL, C-30, and 2,3-DCA are shown as light blue, green, cyan, and magenta, respectively. (**B**) Close-up view of the superposed structures of C6-HSL (green) and cross-docked (cyan) 2,3-DCA in the autoinducer-binding cavity of CviR. (**C**) Close-up view of 2,3-DCA bound to CviR; CviR receptor protein is indicated in blue, hydrogen bonds are shown as yellow dotted lines, amino acid residues of binding sites are shown in violet. (**D**) Key hydrophobic interactions that were predicted to occur between 2,3-DCA and CviR. The images (**A**–**C**) were rendered using the PyMOL Molecular Graphics System, v1.5. The image (**D**) was rendered using LigPlot+ ligand–protein interaction diagrams, v.2.2.

Subsequently, molecular docking studies were performed to analyze the possibility of 2,3-DCA binding interactions with QS receptor protein CviR. The docking energy of 2,3-DCA with CviR as well as the hydrogen bond binding and hydrophobic interactions were investigated using the interactions of the natural ligands C6-HSL with CviR as control and comparing the interaction between the positive control C-30 and CviR. The PyMOL simulation results for hydrogen bond binding and hydrophobic interactions are shown in Figure 7 and Figure S5, and docking energy and amino acid residues involved in the interactions are shown in Table 1. The interactions between C6-HSL and CviR (Figure S5A,a) were highly consistent with previous reports [6,32], providing credibility to the docking method. Moreover, it was found that C-30 could successfully bind to the active-site cavity of CviR (Figure S5B,b), which was similar to that of C-30 bound to LasR at the ligand-binding site in *Pseudomonas aeruginosa* [33]. Compared with the natural ligand C6-HSL, 2,3-DCA was observed to bind to the same receptor pocket of CviR (Figure 7A), but it required higher energy for docking with CviR (Table 1). The disparity in binding sites and interaction modes results in differences in both hydrogen bonding and hydrophobic interaction, and the latter may serve as an important reason contributing to the higher docking energy of 2,3-DCA to CviR compared to that of C6-HSL to CviR.

The process of QS can be disrupted by mainly four different mechanisms: inhibition of biosynthesis of autoinducer molecules, enzymatic inactivation or degradation of the autoinducer, interference with the signal receptor, and inhibition of the genetic regulation system [3,30,34]. In this study, exogenous signal molecules and QS inhibitors were added, and apart from *C*. *violaceum* CV026, there were no other enzymes present. Therefore, QS inhibition of 2,3-DCA in *C*. *violaceum* CV026 did not involve the production and degradation of the QS signal molecule C6-HSL. Additionally, the molecular docking results revealed that both 2,3-DCA and C6-HSL could bind to the same receptor pocket of CviR, indicating that 2,3-DCA inhibited QS probably by competing with signaling molecules for the binding sites of receptor proteins. Combined with qRT-PCR results showing that 2,3-DCA inhibited the expression of QS and QS-related genes *cviR*, *vioA*, *vioB*, and *vioE*, we can postulate that 2,3-DCA inhibited the expression of the signal molecular receptor protein cviR and competitively bound to the active receptor pocket of cviR protein, forming inactive complexes, preventing the normal transcription of downstream genes (*vioA*, *vioB*, and *vioE*), and thus blocking the disease-causing behaviors regulated by QS. Via this mechanism of action, 2,3-DCA inhibited the QS system in *C*. *violaceum*. 

Additionally, other two natural cinnamic acid derivatives, 4-methoxycinnamic acid (MCA) and 4-(dimethylamino) cinnamic acid (DCA), were identified as potential QS and biofilm inhibitors in *C*. *violaceum* ATCC12472 [35,36]. We also analyzed the possibility of MCA (PubChem CID: 699414) and DCA (PubChem CID: 1540638) binding interactions with QS receptor protein CviR by molecular docking studies. Interestingly, compared with 2,3-DCA, both MCA and DCA bound to the same receptor pocket of CviR with partially identical amino acid residues (Figure S6, Table S1), indicating that active cinnamic acid derivatives interfere with CviR and disrupt the essential conformational changes necessary for proper protein folding, thus causing CviR to malfunction and likely leading to its aggregation and proteolytic degradation. These findings also add to the relevant scientific literature indicating that cinnamic acid derivatives hold promise as a potential strategy for treatment against *C*. *violaceum*.

## 3. Materials and Methods

### 3.1. Bacterial Strain and Culture Condition

*N. mentallicus* SCSIO 53858 (Genbank No. SUB14163607) was isolated from a deep-sea sediment sample collected from the South China Sea (118°48′01″ E, 18°17′37″ N) at a depth of 4000 m. The strain was deposited at the RNAM Center for Marine Microbiology, South China Sea Institute of Oceanology, Chinese Academy of Sciences. Each isolate was cultured in marine broth 2216 (MB, BD Difco).

The bacterial strain *C*. *violaceum* CV026 was purchased from the Guangdong Provincial Center for Microbial Strains (Guangzhou, China). The bacterial strain was stored at −80 °C in a refrigerator until use. Each isolate was cultured in LB medium (Hopebio).

### 3.2. Fermentation and Extraction of N. mentallicus SCSIO 53858

A two-stage culturing procedure was employed to grow the *N. mentallicus* SCSIO 53858. *N. mentallicus* SCSIO 53858 was inoculated into 500 mL Erlenmeyer flasks containing 200 mL of MB medium and cultured at 28 °C for 3 days on a thermostatic shaker at 180 rpm. Subsequently, the broth culture (5%) was transferred into the fermentation medium comprising 80 g of rice, 3.6 g of sea salt, and 120 mL distilled water in a 1 L Erlenmeyer flask, and *N. mentallicus* SCSIO 53858 was cultured at 28 °C for 30 days. A total of 185 bottles of culture medium (180 g/bottle on average) were extracted with 70% (*v*/*v*) MeOH-H_2_O and ethyl acetate (3 × 30 L) to generate a crude extract (17.6 g).

### 3.3. Isolation and Structural Elucidation of 2,3-DCA

The crude extract was separated by middle-pressure liquid chromatography (MPLC) on an ODS column (40–60 µm, Agela Technologies, Tianjin, China) using a stepped gradient elution of MeOH-H_2_O (10:90, 20:80, 30:70, 40:60, 50:50, 60:40, 70:30, 80:20, 90:10, 100:0) for separation into seven fractions (Fr.1 to Fr.7) based on thin-layer chromatography properties [37]; each fraction was tested by the violacein assay. Fr.4 (1.0 g) with anti-QS activity was separated by MPLC on a silica gel column using a stepped gradient elution of CH_2_Cl_2_-MeOH (from 100:0 to 0:100) to obtain eight subfractions (Fr.4.1–Fr.4.8). Then, Fr.4.4 (233.7 mg) was further purified by semi-preparative HPLC using an ODS column (YMC-pack ODS-A, 10 × 250 mm, 5 µm, 3 mL/min) with an isocratic elution of 55% (*v*/*v*) MeOH-H_2_O to yield one white amorphous powder compound (27.7 mg). This compound was identified as 2,3-dimethoxycinnamic acid, and its structure was elucidated based on NMR spectra and mass spectrometry spectroscopy. ^1^H, ^13^C NMR, and distortionless enhancement by polarization transfer (DEPT) spectra were recorded on an AVANCE III HD 700 (Bruker, Billerica, MA, USA) instrument. Electrospray ionization MS spectra data were recorded on a MaXis quadrupole time-of-flight mass spectrometer.

### 3.4. QS Inhibitory Activity Assay

The disc diffusion assay was used to screen for the anti-QS active fractions and compounds. First, 15 mL LB agar plates were flooded with 1 mL of an overnight culture of *C*. *violaceum* CV026 in LB broth (OD_600_ = 1.0) and C6-HSL at a concentration of 500 nM to prepare the agar plate as a lawn. Then, 6 mm diameter wells were bored in each agar plate using a flame-sterilized glass tube, and the compounds dissolved in MeOH were added to the wells. Furanone C-30 dissolved in MeOH and MeOH were used as the positive and negative control, respectively. These plates were then incubated at 30 °C for 18 h. The inhibition of QS in *C*. *violaceum* CV026 is manifested as the inhibition of purple pigmentation around the wells containing the compound.

### 3.5. Determination of MIC and Growth Curves

The MIC of 2,3-DCA against the *C*. *violaceum* CV026 strain was determined by broth microdilution as per the Clinical and Laboratory Standards Institute [38], with minor modifications. The strain was inoculated in 3 mL of LB broth medium for 18 h. The OD_600_ was adjusted to 0.7, and this culture was diluted 1000 times to reach an initial bacterial concentration of 1 × 10^6^ CFU/mL [39]. 2,3-DCA was then added, and the mixtures were cultured at 28 °C and 150 rpm for 24 h. MeOH was used as a negative control. For each treatment, 200 μL (at each concentration) was added to 96-well plates (Costar™ 3599, Corning Inc., Corning, NY, USA), which were then cultured at 28 °C and 150 rpm for 24 h. Absorbance was measured at 600 nm (OD_600_) using a microplate reader (BIO-DL K3 Plus, Shanghai, China). Three replicates were performed for each experiment.

### 3.6. Violacein Assay

The effects of 2,3-DCA on violacein production were quantified as previously described with minor modifications [32]. Overnight *C*. *violaceum* CV026 culture was added to LB broth (1:1000, *v*/*v*) supplemented with C6-HSL and 2,3-DCA (50, 100, and 150 µg/mL). MeOH was used as the negative control. Following incubation at 28 °C with shaking overnight, 1 mL of culture was centrifuged for 10 min and 4 °C at 10,000 rpm to obtain insoluble violacein and cells. The bottom precipitated bacteria were added to 1 mL of dimethyl sulfoxide (DMSO) and then thoroughly vortexed for a few minutes and centrifuged for 10 min and 4 °C at 10,000 rpm to remove the insoluble cells. Absorbance was determined at 585 nm using the microplate reader. Three replicates were performed for each experiment. Values are presented as the mean ± SD; *n* = 3.

### 3.7. Swarming Motility Assay

A swarming motility assay was conducted with the method described earlier [40]. First, 1 µL of the above bacterial culture was inoculated in a swarming agar medium consisting of 1% peptone, 0.5% NaCl, 0.5% glucose, and 0.5% agar. The plates were then incubated at 28 °C in an upright position for 24 h. The reduction in swarming migration was recorded by measuring the swarm zones of the bacterial cells after 24 h.

### 3.8. Biofilm Assay

The anti-biofilm potential of 2,3-DCA was determined using the method described previously with minor modifications [35]. Overnight *C*. *violaceum* CV026 culture was added to LB broth (1:1000, *v*/*v*) supplemented with C6-HSL and 2,3-DCA (50, 100, and 150 µg/mL); MeOH was used as the negative control. Then, 200 μL of each sample was added to 96-well polystyrene plates and incubated at 28 °C for 24 h. After 24 h of cultivation, the suspensions were removed, and the wells were washed three times with phosphate-buffered saline (PBS, pH 7.2). The bottom biofilms were fixed with 300 μL of methanol for 15 min and then dried at 60 °C. The dried biofilms were stained with 50 μL of 0.1% crystal violet (*w*/*v*) for 15 min. The wells were washed three times with distilled water to remove residual crystalline purple dye and then dried at 60 °C. After solubilization of the dye with ethanol, the biofilms were quantified by reading the microplates at 570 nm.

SEM analysis was performed based on a previous study [35] with some modifications. SEM analysis was carried out on the biofilms formed on glass coverslips (0.2 mm thick, 12 mm diameter) in 24-well polystyrene plates (Costar™ 3524, Corning Inc., Corning, NY, USA). A bacterial suspension supplemented with 2,3-DCA (50, 100, and 150 μg/mL), or MeOH (negative control) was added to each well, followed by incubation for 24 h at 28 °C. After 24 h of biofilm formation, the suspensions were removed, and the wells were washed with sterile PBS (pH 7.2). We then added 1 mL of fresh LB medium with 2,3-DCA (50, 100, and 150 μg/mL) to the wells. The plates were incubated at 28 °C for 24 h and 150 rpm. The biofilms formed on the coverslips were then washed three times with PBS (pH 7.2) and fixed with 4% glutaraldehyde diluted in 0.1 M PBS (pH 7.2) at 4 °C overnight. The coverslips were again washed three times with PBS (pH 7.2) for 15 min, followed by gradient elution (30%, 50%, 70%, 80%, 90%, 100%) with ethanol for 20 min. After 5 h of freeze drying, the samples were coated with gold. Images were acquired by SEM (JSM6360, JEOL, Tokyo, Japan).

### 3.9. qRT-PCR Analysis

qRT-PCR was performed based on a previous study [41], with some modifications. The primers used for qRT-PCR are listed in Table S2. Total RNA was extracted from the MeOH and 2,3-DCA (150 μg/mL) groups of bacterial cells using an RNA extraction kit (Transgen Biotech, Beijing, China) according to the manufacturer’s instructions. Total RNA was treated with gDNA wiper mix reagent to remove genomic DNA, and cDNA was synthesized using the HiScript^®^ III 1st Strand cDNA Synthesis Kit (+gDNA wiper, Vazyme Biotech, Nanjing, China). qRT-PCR amplification was performed by ChamQ Blue Universal SYBR qPCR Master Mix (Vazyme Biotech, Nanjing, China) in a CFX Connect Real-Time PCR Detection System (BIO-RAD, Hercules, CA, USA), as per the manufacturer’s protocols. The thermocycling parameters were as follows: 3 min at 95 °C; 40 cycles of 10 s at 95 °C, 30 s at 52 °C. All reactions were run in triplicate. The results obtained were normalized using threshold cycles for the endogenous control (16S rRNA). Relative expression levels of the genes were calculated using the 2^−ΔΔCt^ method. Experiments were performed in triplicate using different RNA samples each time.

### 3.10. Molecular Docking Studies

All computations were carried out by a series of software programs including MGLTools 1.5.7, PyMol 1.5.0.3, Autodock 4.2.6, and LigPlot+ v.1.4 [42,43,44]. The natural ligand C6-HSL, positive control furanone C-30, and studied molecule 2,3-DCA in SDF format were downloaded from the PubChem database with the compound CID 10058590, 10131246, and 735842, respectively. The crystal structure of the *C*. *violaceum* CviR receptor protein bound to the native ligand C6HSL was downloaded from the protein data bank (PDB) with the ID code 3QP1 [6]. The water molecules and co-crystallized ligand of 3QP1 were first eliminated. Next, the protein was modified by adding polar hydrogens and computing Gasteiger charges. The grid box was then settled to the whole receptor involved in the active site, where the number of points in x-, y-, and z-dimension was 126, the spacing (angstrom) was 0.403, and the docking parameter files were created using the Lamarckian genetic algorithm (GA) before running the program. The number of GA runs was set to 100 replications, and the other settings were set as defaults. The results of the docking computations were ranked by binding energy. The conformations and interactions between the ligand and the target protein were visualized by the PyMol molecular graphic system and LigPlot+.

### 3.11. Statistical Analysis

The data were analyzed using SPSS 21.0 software, and the data were edited by GraphPad Prism 9 software. Data represent the means for three independent experiments, and statistical significance is highlighted with asterisks in the figures as follows: *p* > 0.05, not significant; *p* < 0.05 (*), significant; *p* < 0.01 (**), extremely significant.

## 4. Conclusions

In summary, 2,3-DCA was isolated and identified from the crude extract of the marine actinomycete *N*. *mentallicus* SCSIO 53858 and exhibited anti-QS activity with the effects of inhibiting violacein, biofilm formation, and swimming motility on model bacteria *C*. *violaceum* CV026. Furthermore, qRT-PCR and molecular docking results revealed that 2,3-DCA exerts its anti-bacterial pathogenicity effects via inhibiting the expression of QS and QS-related genes *cviR*, *vioA*, *vioB*, and *vioE* and suppressing the QS system by competing with the natural signal molecule C6-HSL for binding to the same pocket of the CviR receptor. The demonstrated capacity of 2,3-DCA to weaken pathogenic bacteria suggests its potential to reduce the likelihood of resistance development. This makes 2,3-DCA a promising candidate for the development of a novel strategy to combat drug-resistant *C*. *violaceum* infections.

## Figures and Tables

**Table 1:** Details of the docked complex of the CviR receptor protein with the natural ligand C6-HSL, C-30, and 2,3-DCA.

Molecule	Binding Energy (kcal/mol)	Hydrogen Bonding Interactions	Key Hydrophobic Interactions
C6-HSL	−7.53	Asp97, Tyr80, Trp84, Ser155	Ile99, Trp111, Tyr88, Leu85, Phe126, Met135, Leu57, Phe115, Ala130
C-30	−6.08	Trp84	Tyr88, Tyr80, Trp84, Leu85, Asp97
2,3-DCA	−5.63	Asp97, Met135, Ser155	Phe115, Trp111, Tyr80, Tyr88, Leu57, Trp84, Ile153, Ile99, Leu100, Leu85, Phe126

## Data Availability

The data presented in this study are available in this article and Supplementary Materials.

## Supplementary Materials

The following supporting information can be downloaded at https://www.mdpi.com/article/10.3390/md22040177/s1: Figure S1: Phylogenetic dendrogram of *Nocardiopsis mentallicus* SCSIO 53858 on the basis of 16S rRNA gene sequences; Figure S2: The ^1^H-NMR spectrum of the obtained compound in methanol-*d*_4_; Figure S3: The ^13^C-NMR spectrum of the obtained compound in methanol-*d*_4_; Figure S4: Illustration of the violacein biosynthesis; Figure S5: Docked complex of the CviR receptor protein with natural ligand C6-HSL and C-30; Figure S6: Docked complex of the CviR receptor protein with 4-methoxycinnamic acid (MCA) and 4-(dimethylamino) cinnamic acid (DCA); Table S1: Details of the docked complex of the QS receptor protein CviR with MCA and DCA; Table S2: PCR primers for qRT-PCR. “Reference [30] is also cited in the supplementary materials”.
