# Supplementary material for: 2,3-Dimethoxycinnamic Acid from a Marine Actinomycete, a Promising Quorum Sensing Inhibitor in Chromobacterium violaceum
Source: Mar Drugs. 2024 Apr 16;22(4):177. doi: 10.3390/md22040177 (PMC11051081; doi:10.3390/md22040177)
Supplement: Supplementary file 1 [file marinedrugs-22-00177-s001.zip › marinedrugs-2926567-supplementary.pdf]

Supplementary materials

2,3-dimethoxycinnamic acid from marine actinomycete, a promising quorum sensing inhibitor in *Chromobacterium violaceum*

Yanqun Li [1,3], Wenping Ding [1], Jiajia Yin [1,3], Xingyu Li [1], Xinpeng Tian [1,2,4], Zhihui Xiao [1], Fazuo Wang [1] and Hao Yin [1,2,3,4,*]

[1] CAS Key Laboratory of Tropical Marine Bio-Resources and Ecology, South China Sea Institute of Oceanology, Chinese Academy of Sciences, Guangzhou 510301, China; liyanqun20@mails.ucas.ac.cn (Y.L.); dingwenping19@mails.ucas.ac.cn (W.D.); yinjiajia22@mails.ucas.ac.cn (J.Y.); bsy_lixingyu@126.com (X.L.); xinpengtian@scsio.ac.cn (X.T.); xzh@scsio.ac.cn (Z.X.); wangfazuo@scsio.ac.cn (F.W.)

[2] Southern Marine Science and Engineering Guangdong Laboratory (Guangzhou), Guangzhou 511458, China

[3] University of Chinese Academy of Sciences, Beijing 100049, China

[4] Sanya Institute of Ocean Eco-Environmental Engineering, Sanya, 572025, China

[*] Correspondence: yinhao@scsio.ac.cn (H.Y.) Tel.: +86-15919668007 or +86-020-89023103

Abstract: An ethyl acetate extract of a marine actinomycete strain, *Nocardiopsis. mentallicus* SCSIO 53858, isolated from a deep-sea sediment sample in the South China Sea, exhibited anti-quorum sensing (QS) activity against *Chromobacterium violaceum* CV026. Guided by the anti-QS activity, a novel active compound was isolated and purified from the extract, and was identified as 2,3-dimethoxycinnamic acid (2,3-DCA) through spectral data analysis. At a concentration of 150 μg/mL, 2,3-DCA exhibited robust inhibitory effects on three QS-regulated traits of *C. violaceum* CV026: violacein production, swarming motility, and biofilm formation, with inhibition rates of 73.9%, 65.9%, and 37.8%, respectively. The qRT-PCR results indicated that 2,3-DCA can disrupt QS system in *C. violaceum* CV026 by effectively suppressing the expression of QS-related genes, including *cviR*, *vioA*, *vioB*, and *vioE*. Molecular docking analysis revealed that 2,3-DCA hinders the QS system by competitively binding to the same binding pocket on the CviR receptor as the natural signal molecule C6-HSL. Collectively, these findings suggest that 2,3-DCA exhibits promising potential as an inhibitor of QS systems, providing a potential solution to the emerging problem of bacterial resistance.

Keywords: *Nocardiopsis metallicus* SCSIO 53858; quorum sensing inhibitor; 2,3-dimethoxycinnamic acid; *Chromobacterium violaceum*; molecular docking

Content

Figure S1. Phylogenetic dendrogram of *Nocardiopsis mentallicus* SCSIO 53858 on the basis of 16S rRNA gene sequences. 2
Figure S2. The ^{1}H-NMR spectrum of the obtained compound in methanol-d_4. 3
Figure S3. The ^{13}C-NMR spectrum of the obtained compound in methanol-d_4. 3
Figure S4. Illustration of the violacein biosynthesis [1] 4
Figure S5. Docked complex of the CviR receptor protein with natural ligand C6-HSL and C-30. 5
Figure S6. Docked complex of the CviR receptor protein with 4-methoxycinnamic acid (MCA) and 4-(dimethylamino) cinnamic acid (DCA) 6

Table S1. Details of the docked complex of the QS receptor protein CviR with MCA and DCA. 6
Table S2. PCR primers for qRT-PCR. 7

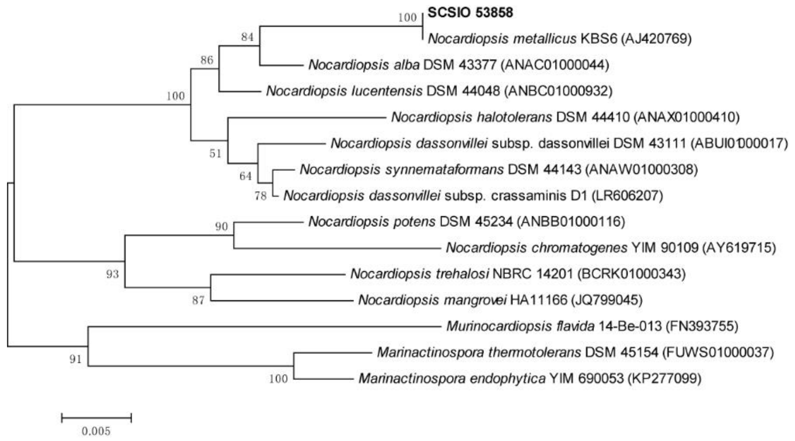

Figure S1. Phylogenetic dendrogram of *Nocardiopsis mentallicus* SCSIO 53858 on the basis of 16S rRNA gene sequences. Scale bar: 0.005 nucleotide substitution per 200 nucleotides of 16S rRNA sequence; Numerals on branches are the supporting percentage by 1000 replicates.

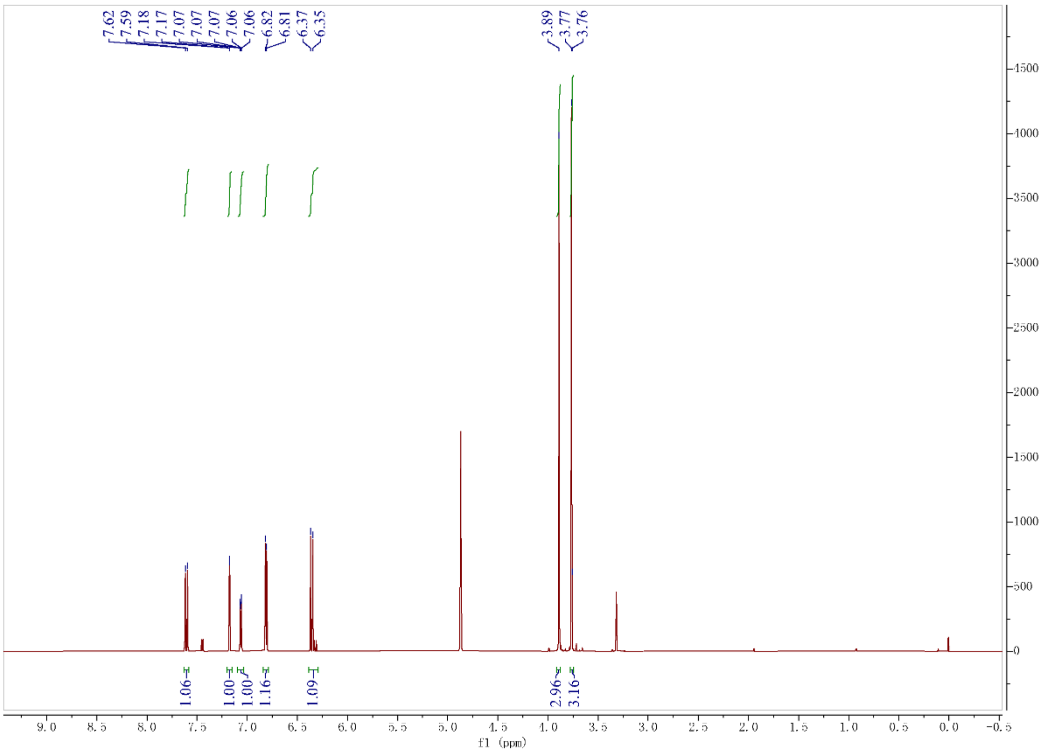

Figure S2. The ^{1}H-NMR spectrum of the obtained compound in methanol-d_4.

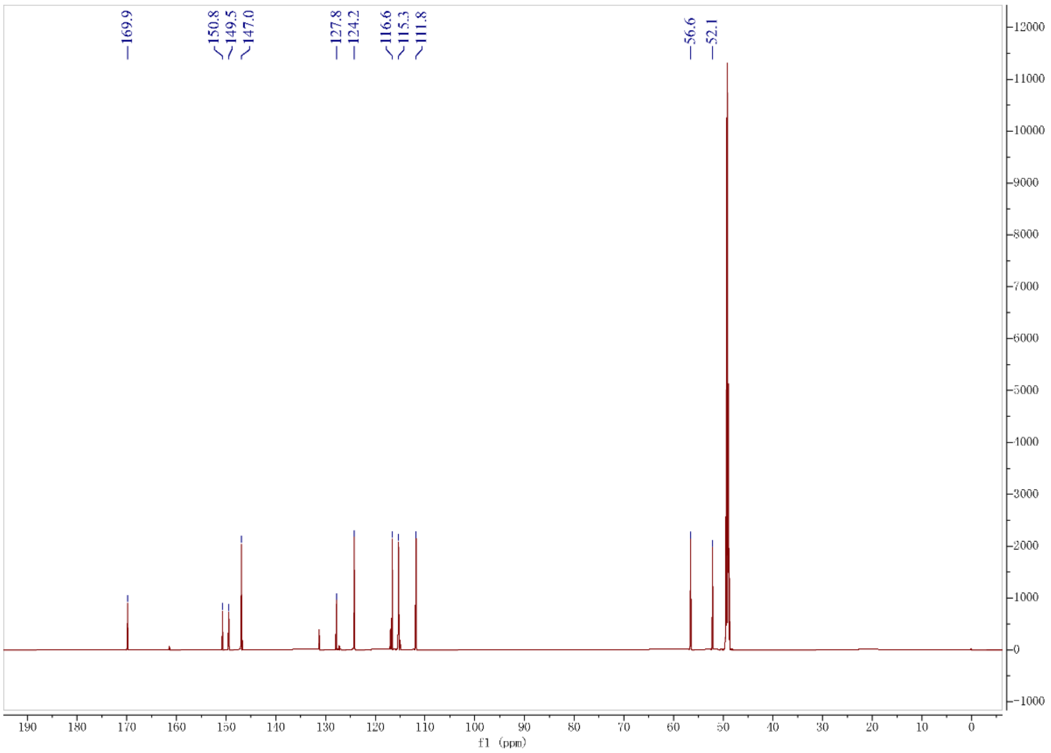

Figure S3. The ^{13}C-NMR spectrum of the obtained compound in methanol-d_4.

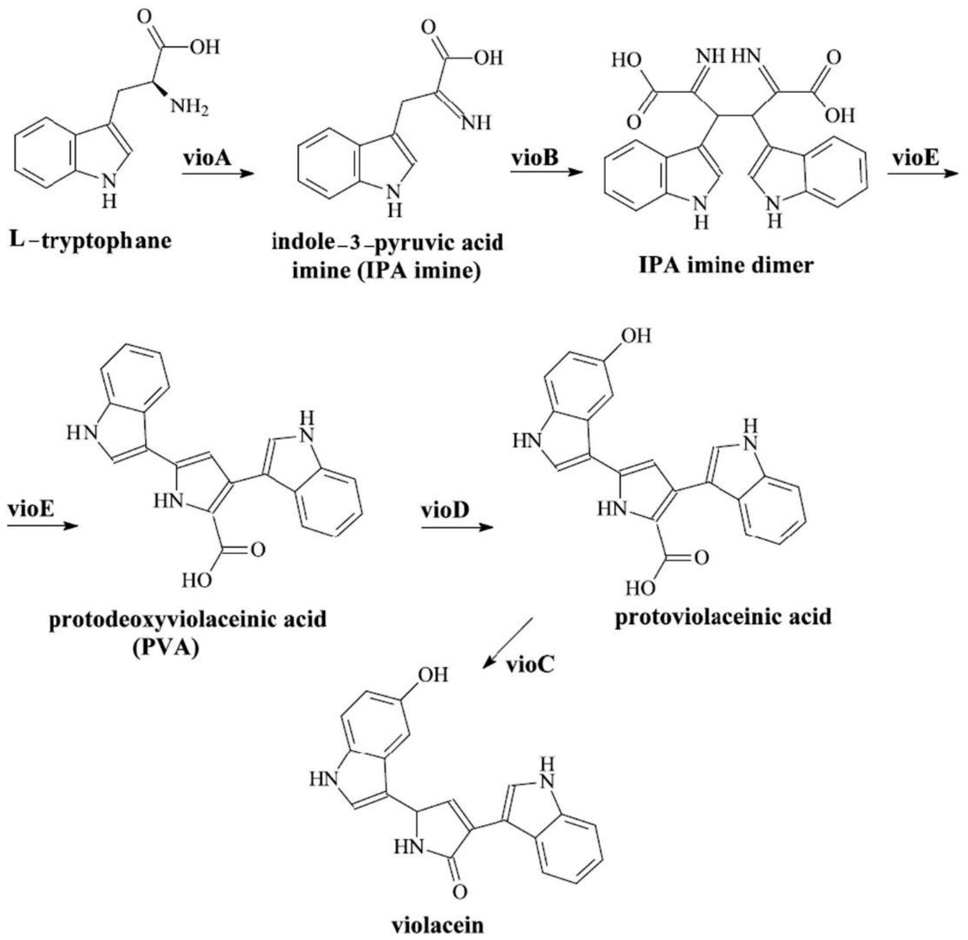

Figure S4. Illustration of the violacein biosynthesis [1]: The enzyme VioA is L-tryptophan oxidase that has FAD as a co-factor and is the first enzyme required for an IPA imine formation. This imine becomes then a substrate for VioB enzyme that forms a dimmer from it. VioB is an oxidase that uses heme moeity as the co-factor. It is believed that the key enzyme in the violacein biosynthetic pathway is VioE, the third enzyme that takes action in the proposed pathway. VioE uses the IPA imine dimmer as a substrate and transforms in to intermediates that are then used as substrates for VioD and VioC enzymes. The last two enzymes are classified as monooxygenases, both being FAD dependent, and responsible for violacein and deoxyviolacein production.

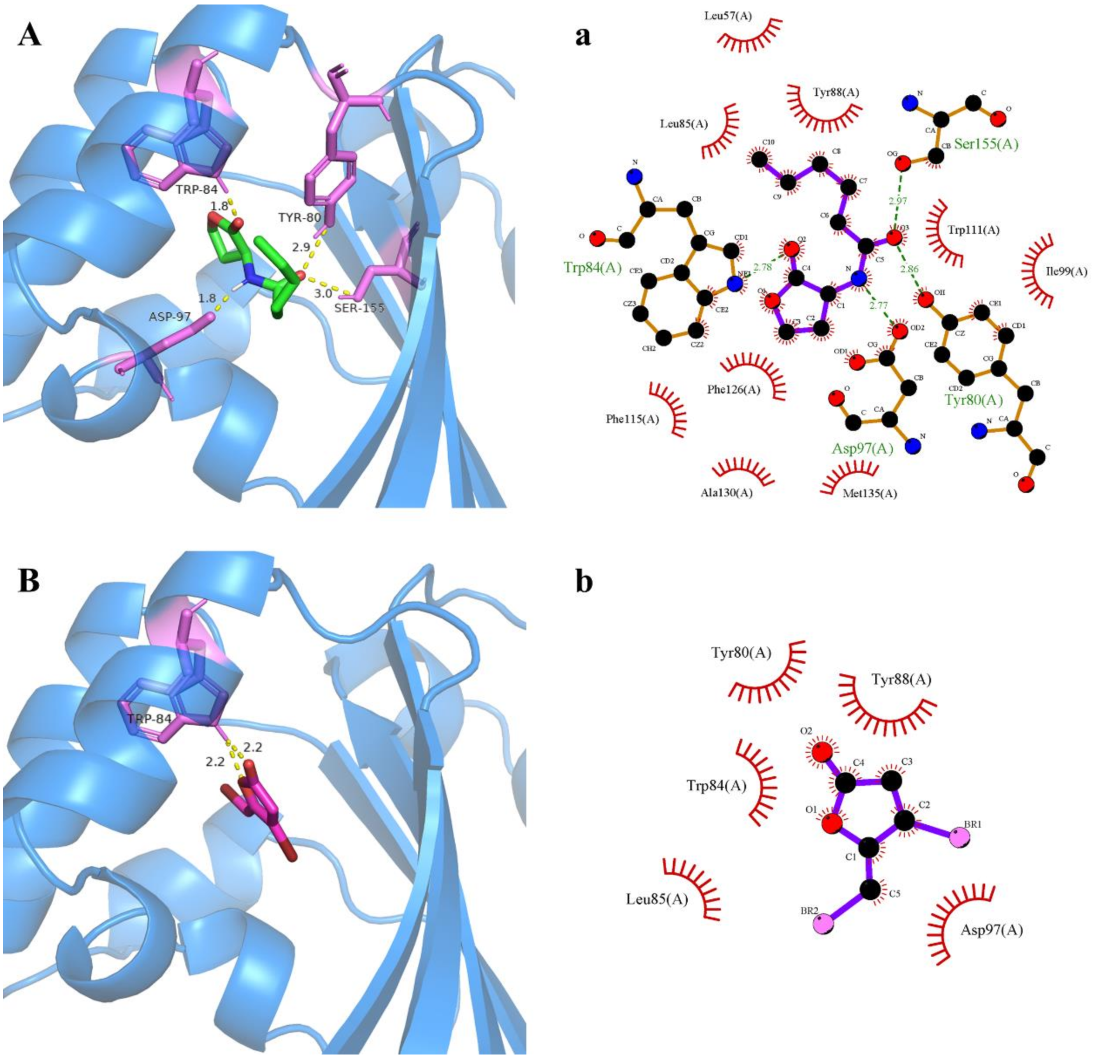

Figure S5. Docked complex of the CviR receptor protein with natural ligand C6-HSL and C-30. (A) C6-HSL (green) bound to CviR. (B) C-30 (magenta) bound to CviR. CviR receptor protein was indicated in blue, the hydrogen bonds were shown as yellow dotted lines, the amino acid residues of binding sites were shown in violet. (a) LigPlot of C6-HSL bound to CviR, showing the key hydrophobic interactions. (b) LigPlot of C-30 bound to CviR, showing the key hydrophobic interactions.

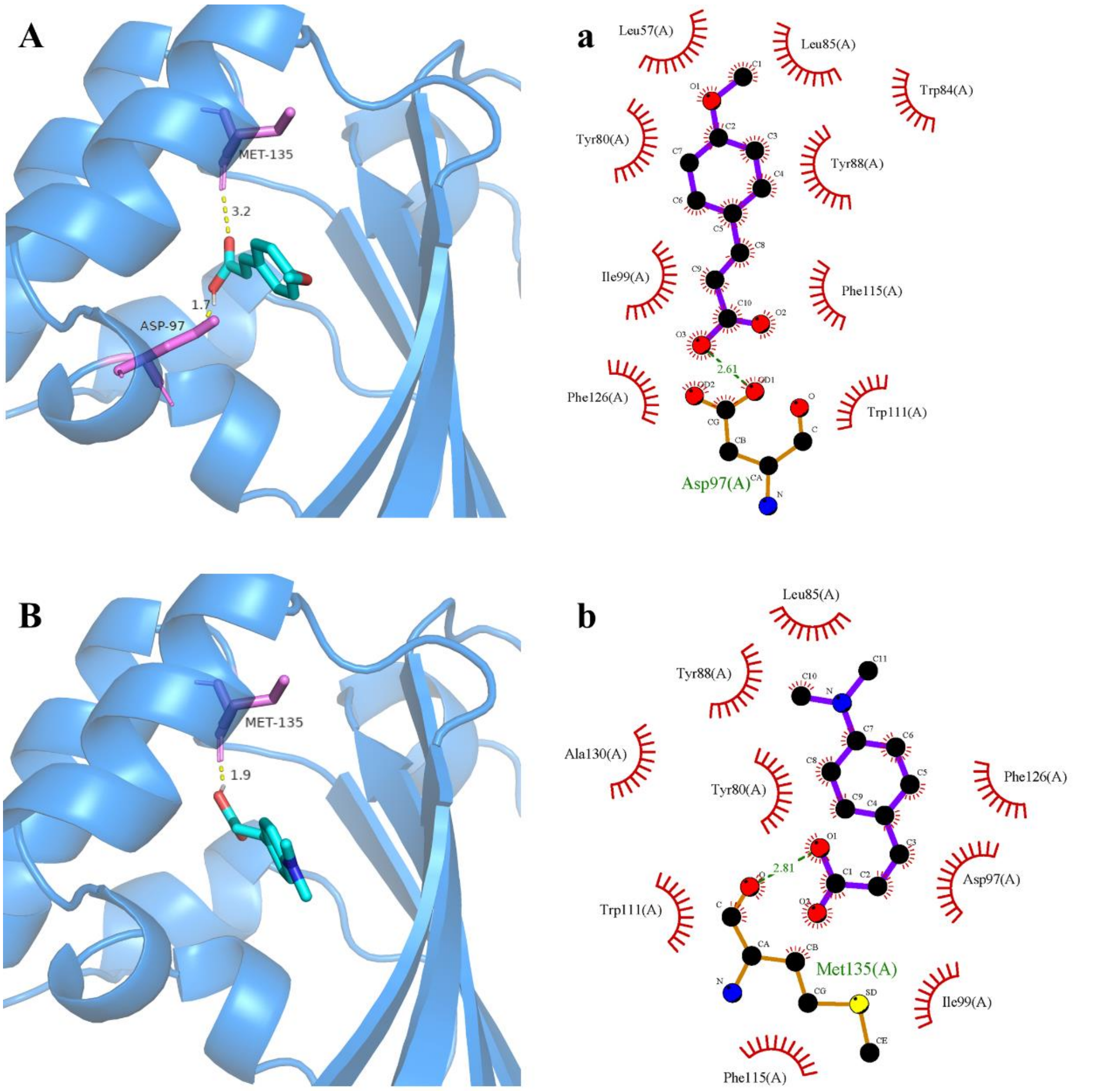

Figure S6. Docked complex of the CviR receptor protein with 4-methoxycinnamic acid (MCA) and 4-(dimethylamino) cinnamic acid (DCA). (A) 4-methoxycinnamic acid bound to CviR. (B) 4-(dimethylamino) cinnamic acid bound to CviR. CviR receptor protein was indicated in blue, the hydrogen bonds were shown as yellow dotted lines, the amino acid residues of binding sites were shown in violet. (a) LigPlot of 4-methoxycinnamic acid bound to CviR, showing the key hydrophobic interactions. (b) LigPlot of 4-(dimethylamino) cinnamic acid bound to CviR, showing the key hydrophobic interactions.

Table S1. Details of the docked complex of the QS receptor protein CviR with MCA and DCA.

Molecule	Binding energy (kcal/mol)	Hydrogen bonding interactions	Key hydrophobic interactions
MCA	-5.43	Asp97, Met135	Leu57, Tyr80, Ile99, Phe126, Trp111, Phe115, Tyr88, Leu85, Trp84
DCA	-6.07	Met135	Leu85, Tyr88, Tyr80, Trp111, Phe115, Ile99, Asp97, Phe126, Ala130

Table S2. PCR primers for qRT-PCR.

Gene	Function	Sequence (5'-3')	Product size (bp)
*vio*A	Flavin-dependent tryptophan 2-mono-oxygenase	F: TCTGTCTTATGACGAGCCG<br>R: CCAGTAAGCCGCCATTT	142
*vio*B	Considered to be a polyketide synthase, containing heme protein	F: GAGATAGCCCACGAGGAGATG<br>R: CGGCGAAGGAATGAACG	210
*vio*C	FAD-dependent monooxygenase	F: CGTCCGCTACCACTTCG<br>R: TGGGCGTGCCTTCATAC	365
*vio*D	Flavin-dependent monooxygenase	F: CGGCTAATCCGCTGTCC<br>R: AGTGCGAAGTCTTGTGGTTG	282
*vio*E	Responsible for conversion of flavanone to isoflavone	F: TCGGGCTATTGCTGGTT<br>R: TTCTGCTTGCGGGTGC	143
*cvi*R	LuxR family transcriptional regulator	F: TCGCAGCAATGGACTTT<br>R: CGAGATGGATTCGCAGTA	259
16S rRNA	Housekeeping gene	F: CAGAAGGGCGTGGAGATG<br>R: GCGTCAGGATGGGCAGTA	178

References

1. Durán, N.; Justo, G. Z.; Durán, M.; Brocchi, M.; Cordi, L.; Tasic, L.; Castro, G. R.; Nakazato, G., Advances in Chromobacterium violaceum and properties of violacein-Its main secondary metabolite: A review. *Biotechnology Advances* **2016,** 34, (5), 1030-1045.
